# Variations in societal characteristics of spatial disease clusters: examples of colon, lung and breast cancer in Japan

**DOI:** 10.1186/1476-072X-4-16

**Published:** 2005-06-14

**Authors:** Yoshiharu Fukuda, Masahiro Umezaki, Keiko Nakamura, Takehito Takano

**Affiliations:** 1 Health Promotion/International Health, Division of Public Health, Graduate School of Tokyo Medical and Dental University, Yushima 1-5-45, Bunkyo, Tokyo, 113-8519, Japan

## Abstract

**Background:**

Spatial analyses and ecological studies are essential for epidemiology and public health. The present study combining these two methods was performed to identify spatial clusters of selected types of cancer in Japan and to determine their societal characteristics focusing on homogeneity among clusters.

**Results:**

Spatial clusters of high mortality rates of male colon and lung cancer and of female breast cancer were identified by the spatial scan statistic using Japanese municipal data (N = 3360) from 1993 to 1998 and also municipalities were divided into four societal clusters based on socioeconomic indicators and population density (urban-rich, suburban, rural-poor, and clutter). Five, seven, and four mortality clusters were identified for lung, colon and breast cancer, respectively. For colon and breast cancer, most municipalities of all except one cluster were included in a single societal cluster (urban-rich). The municipalities associated with mortality clusters for lung cancer belonged to various societal clusters.

**Conclusion:**

Increased mortality rates of colon and breast cancer can be explained by homogenous societal characteristics related to urbanisation, although there were exceptional areas with higher mortality rates. The regional variation in lung cancer mortality rate appeared to be due to heterogeneous factors. These findings and the analysis performed in the present study will contribute to both nationwide and region-specific cancer prevention strategies.

## Background

Health levels vary substantially between different regions, and it is essential to characterise these regional variations and identify areas with an accumulation of health problems for epidemiologic research and to allow appropriate public health policy decisions [1,2]. Recent advancements in technologies, such as geographic information systems (GIS), have allowed the application of not only disease mapping but also spatial analyses, such as spatial clustering and cluster detection, in epidemiological research [3-6]. In this context, clusters are defined as unusual concentrations of health events in both space and time [1].

Ecological studies examining the relationships between regional health levels and various characteristics represent another essential approach in epidemiology and public health. Although such studies have a number of limitations, especially confounding factors and ecological fallacy, factors that may contribute to regional health variations can be identified and hypotheses can be formulated for further research [7,8]. Several ecological studies have demonstrated relationships between mortality and regional characteristics related to the environment, health-related behaviour, and economic and demographic factors in Japan [9-12]. Recent systematic studies using municipal data regarding all causes and cause-specific mortality along with large numbers of societal indicators showed substantial relationships between a region's mortality rate and societal characteristics [13-15].

In general, the relations between health levels and regional characteristics are examined by correlation and regression analyses [8-14]. These methods can reveal factors correlated with regional variations in a specific health issue across study areas. However, if various factors contribute separately to a health issue for different areas, or where there is an exceptional factor contributing to a health issue in a limited area, such analyses would not be effective in identifying the contributing factors and may overlook exceptional factors.

The present study was performed to determine whether areas with a specific health problem have homogeneous regional characteristics or different patterns of characteristics. We first identified spatial clusters of three common types of cancer (colon, lung and breast) using the spatial scan statistic. The societal characteristics of the clusters were then elucidated, focusing on heterogeneity in the characteristics among clusters, using municipal data across Japan.

## Results

The result of principle component analysis for seven socioeconomic indicators, to reduce the number of variables and identify dimensional societal factors, was shown in Table 1. Two principle components were identified and they accounted for 78.1% of the total variance inherent in the data. The meaning of these components was considered higher unemployment and overcrowding for the first component and higher income and educational level for the second component. Component scores of the components were assigned to each municipality as societal indices, designated Index 1 and Index 2, respectively, with a mean of 0.0 and standard deviation of 1.0.

**Table 1:** Result of principle component analysis.

Rotated component Matrix from the principle component analysis of seven societal indicators.		
Societal indicator	Component 1	Component 2

Unemployment rate (women)	**0.88**	-0.10
Unemployment rate (men)	**0.87**	0.18
Number of rooms per household	**-0.75**	-0.32
Dwelling area per capita	**-0.77**	-0.28
Education level (women)	0.19	**0.94**
Education level (men)	0.23	**0.91**
Income per capita	0.07	**0.89**

**Figure 1:**
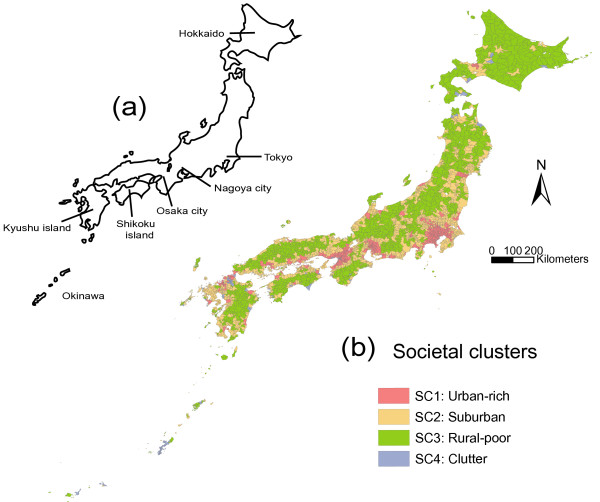
**Distribution of societal clusters**. (a) Map of Japan. (b) Municipalities are classified into four societal clusters (SCs) according to two societal indices (high unemployment and overcorwing and high income and educational level: see Table 1) and popualtion density. The characeritics of clusters are shown in Table 2.

The results of cluster analysis for the purpose of categorization of municipalities into societal cluster (SC) are shown in Table 2. SC1 was characterized with high Index 2 and high population density; SC2 with moderate Index 1, Index 2 and population density; SC3 with low Index 1, Index 2 and population density; and SC4 with high Index 1 and population density and low Index 2. The map of these societal clusters is shown in Figure 1. Most of municipalities in the metropolitan areas such as Tokyo, Nagoya, and Osaka, and most of municipalities of seat of prefectural government belong to SC1. In generally, SC2 are located surrounding SC1, and SC3 are located in mountain areas. SC4 are separately distributed, including some municipalities in Okinawa prefecture and the central part of Osaka. The characteristics of societal clusters were interpreted as urban-rich, suburban, rural-poor, and clutter, respectively.

**Table 2:** Characteristics of societal clusters.

Comparison of societal indices and population density among societal clusters (SC).				
Variable	Societal cluster			
	
	SC1 (N = 507)	SC2 (N = 1483)	SC3 (N = 1246)	SC4 (N = 124)

Index 1: high unemployment and overcrowding	0.89 ± 0.66	-0.08 ± 0.63	-0.54 ± 0.74	2.69 ± 1.23
Index 2: high income and educational level	1.56 ± 1.06	-0.06 ± 0.64	-0.45 ± 0.62	-1.13 ± 0.69
Population density (log)	8.04 ± 0.84	5.75 ± 0.63	3.68 ± 0.83	6.44 ± 1.31

**Figure 2:**
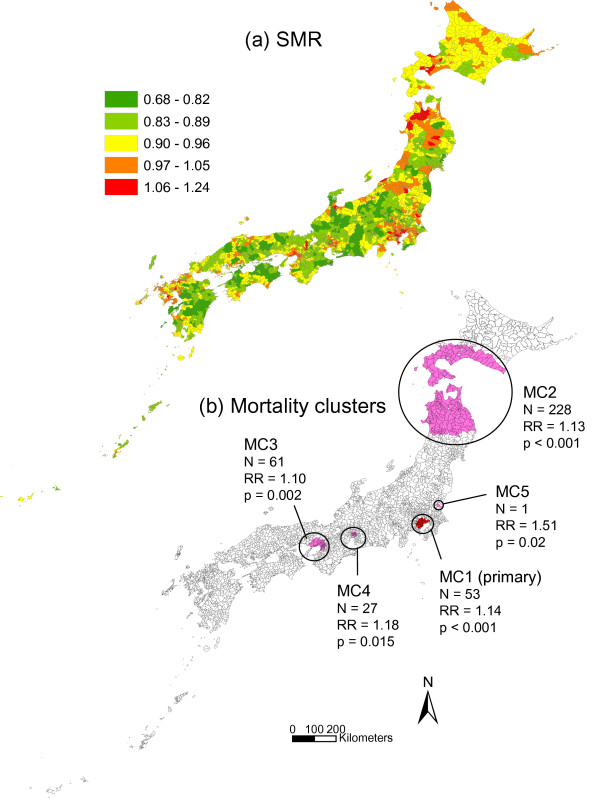
**SMR and mortality clusters for colon cancer**. Municipal standardized mortality ratio (SMR) (a) and mapping of mortality clusters (MC) (b) with higher mortality rates from male colon cancer. The clusters were identified by the spatial scan statistic. N = number of municipalities belonging to each cluster. RR = relative risk.

**Figure 3:**
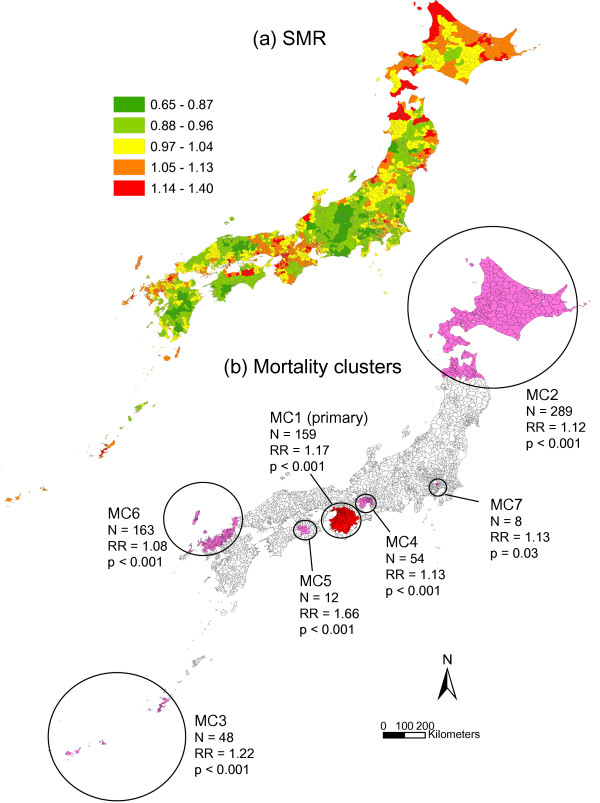
**SMR and mortality clusters for lung cancer**. Municipal standardized mortality ratio (SMR) (a) and mapping of mortality clusters (MC) (b) with higher mortality rates from male lung cancer. The clusters were identified by the spatial scan statistic. N = number of municipalities belonging to each cluster. RR = relative risk.

**Figure 4:**
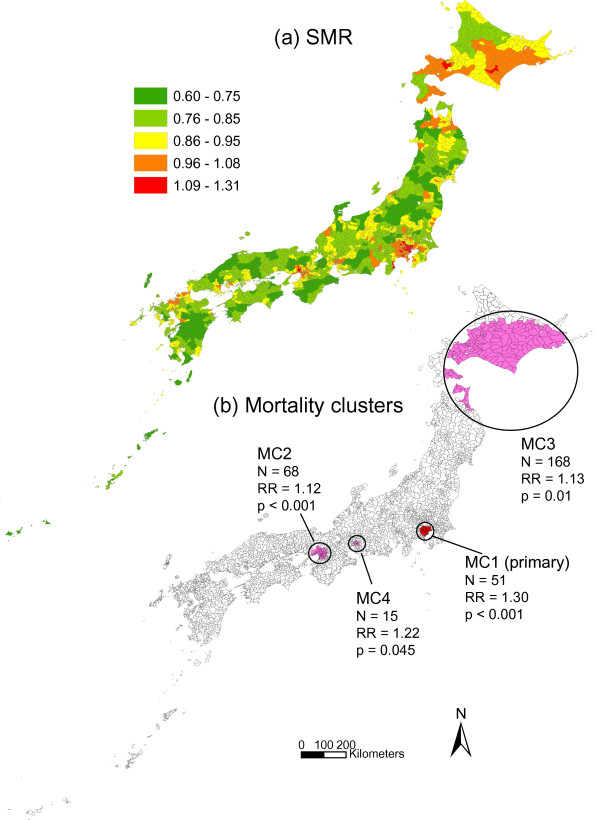
**SMR and mortality clusters for breast cancer**. Municipal standardized mortality ratio (SMR) (a) and mapping of mortality clusters (MC) (b) with higher mortality rates from female breast cancer. The clusters were identified by the spatial scan statistic. N = number of municipalities belonging to each cluster. RR = relative risk.

Municipal standardized mortality ratio (SMR) and the results of spatial scan statistic for male colon and lung cancer and female breast cancer are shown in Figures 2, 3 and 4. As shown in Figure 2 (b), the primary cluster for colon cancer (MC1) included 53 municipalities with a relative risk (RR) of 1.14, and was located in the Tokyo metropolitan area. Four additional clusters were also identified: MC2 was located in the northern part of the main island (Honshu Island) and Hokkaido Island (Hokkaido prefecture), MC3 and MC4 were located in Osaka and Nagoya, which are the second and third largest metropolitan areas after the Tokyo area, respectively, and MC5 that included only one city.

The mortality clusters of male lung cancer are illustrated in Figure 3 (b). The primary cluster (MC1) was located in an area including Osaka, with RR of 1.17. A total of six secondary clusters were also identified. The municipalities of MC2 belonged mainly to Hokkaido prefecture, and those of MC3 belonged to Okinawa prefecture consisting of the southern islands. MC4, MC6, and MC7 included the metropolitan areas of Nagoya, Fukuoka and Tokyo, respectively, while MC5 was located in the mountainous area on Shikoku Island.

As shown in Figure 4 (b), of the four mortality clusters identified for female breast cancer, three were located in metropolitan areas: MC1 in Tokyo, MC2 in Osaka and MC4 in Nagoya. The centre of the remaining cluster, MC3, was located in Hokkaido.

RRs of societal indices, population density, and societal clusters for cancer mortality, which were estimated by the hierarchical Poisson regression, are shown in Table 3. Mortality from colon and breast cancers was significantly and positively related to societal indices and population density. Mortality from lung cancer was significantly and negatively related to Index 2. SC2, SC3, and SC4 showed the lower RR compared to SC1 for colon and breast cancers, while SC4 showed the higher RR for lung cancer.

**Table 3:** Relative risk of societal indices, population density and societal cluster.

Relative risk was estimated by the hierarchical Poisson regression.							
		Male colon cancer		Male lung cancer		Female breast cancer	
		
Variable		Crude	Adjusted ^a^	Crude	Adjusted ^a^	Crude	Adjusted ^a^

Societal Index	Index 1	1.104	1.062	1.055	1.039	1.124	1.077
	Index 2	1.040	1.005*	0.964	0.952	1.097	1.061
Population density (log)		1.064	1.039	1.010	1.017	1.101	1.039
Societal cluster	SC1^b^	1.000	-	1.000	-	1.000	-
	SC2	0.903	-	1.011*	-	0.827	-
	SC3	0.779	-	0.974*	-	0.658	-
	SC4	0.993*	-	1.179	-	0.969	-

^a^The model included societal indices and population density. ^b^The reference for societal clusters. *not significant (p > = 0.05)

Table 4 shows the relationships between mortality clusters and societal clusters. For MC1, MC3, MC4 and MC5 in colon cancer, the dominant societal cluster was SC1. For MC2 the proportion of SC1 was very low (3.5%), and the dominant cluster was SC3 (66.7%). In lung cancer, there were variations in dominant societal clusters. Similar patterns of societal cluster were observed only for MC1 and MC4 with SC1 as the dominant societal cluster and for MC2 and MC5 with SC3. In breast cancer, most municipalities of MC1, MC2 and MC3 consisted of SC1. For MC3, the dominant societal cluster was SC3 (75.0%).

**Table 4:** Relationship between mortality clusters and societal clusters.

Figures show the number of municipalities and the percentage in parenthesis by mortality cluster.				
Mortality cluster ^a^	Societal cluster ^b^			
	
	SC1 (N = 507)	SC2 (N = 1483)	SC3 (N = 1246)	SC4 (N = 124)

Male colon cancer				

MC1 (N = 53)	53 (100.0)			
MC2 (N = 228)	8 (3.5)	59 (25.9)	152 (66.7)	9 (3.9)
MC3 (N = 61)	45 (73.8)	7 (11.5)		9 (14.8)
MC4 (N = 27)	25 (92.6)	2 (7.4)		
MC5 (N = 1)	1(100.0)			

Male lung cancer				

MC1 (N = 159)	81 (50.9)	33 (20.8)	33 (20.8)	12 (4.8)
MC2 (N = 289)	8 (2.8)	46 (15.9)	221 (76.5)	14 (4.8)
MC3 (N = 48)		5 (10.4)	6 (12.5)	37 (77.1)
MC4 (N = 54)	28 (51.9)	24 (44.4)	2 (3.7)	
MC5 (N = 12)		4 (33.3)	7 (58.3)	1 (8.3)
MC6 (N = 162)	27 (16.7)	94 (58.0)	16 (9.9)	25 (15.4)
MC7 (N = 8)	8 (100.0)			

Female breast cancer				

MC1 (N = 51)	51 (100.0)			
MC2 (N = 68)	57 (83.8)	2 (2.9)		9 (13.2)
MC3 (N = 168)	8 (4.8)	24 (14.3)	126 (75.0)	10 (6.0)
MC4 (N = 15)	15 (100.0)			

^a^Mortality cluster (MC) of municipalities with high mortality was identified by the spatial scan statistic: Figures 2-4. ^b^Societal cluster (SC) of municipalities was classified by cluster analysis with two societal indices and population density (see Table 2).

Comparisons of mortality clusters before and after adjustment for societal indices, population density, and societal clusters are shown in Table 5. After adjustment in colon cancer, MC1, MC3, MC4, and MC5 were not detected or showed a decrease of RR. In lung cancer, only MC2 was not detected after adjustment. In breast cancer, MC1, MC2 and MC4 were not detected or showed a decrease of RR, while MC3 showed an increase of RR after adjustment.

**Table 5:** Comparisons of crude and adjusted mortality clustering

Mortality cluster^a^	Crude		Adjusted ^b^		Adjusted ^c^	
	
	RR	p-value	RR	p-value	RR	p-value
Male colon cancer						

MC1	1.14	<0.001	*		1.10	<0.001
MC2	1.13	<0.001	1.14	<0.001	1.15	<0.001
MC3	1.10	0.002	*		*	
MC4	1.18	0.015	*		*	
MC5	1.51	0.02	1.39	0.009	*	

Male lung cancer						

MC1	1.17	<0.001	1.11	<0.001	1.16	<0.001
MC2	1.12	<0.001	1.08	<0.001	1.10	<0.001
MC3	1.22	<0.001	*		*	
MC4	1.13	<0.001	1.12	<0.001	1.08	<0.001
MC5	1.66	<0.001	1.61	0.004	1.65	<0.001
MC6	1.08	<0.001	1.13	<0.001	1.09	0.007
MC7	1.13	0.03	1.08	0.02	1.13	<0.001

Female breast cancer						

MC1	1.30	<0.001	*		1.22	<0.001
MC2	1.12	<0.001	*		*	
MC3	1.13	0.01	1.19	<0.001	1.16	<0.001
MC4	1.22	0.045	*		*	

^a^Mortality cluster (MC) of municipalities with high mortality was identified by the spatial scan statistic: Figures 2-4. ^b^Adjusted for relative risk of societal indices and population density.

^c^Adjusted for relative risk of societal cluster. *Cluster was not detected.

## Discussion

The results of the present study identified spatial clusters with high mortality rates of colon and lung cancer in men, and of breast cancer in women in Japan. The societal characteristics of the municipalities belonging to these clusters were determined by the relationships between mortality clusters and societal clusters. A single dominant societal cluster was detected for colon and breast cancer, although one mortality cluster was exclusive for each cancer. In contrast, we did not detect a dominant societal cluster for lung cancer.

The detection of a single dominant societal cluster for colon and breast cancer, SC1, suggested that there were homogeneous area characteristics for increased mortality due to these types of cancer. This societal cluster had a high Index 2 representing high income and education level and high population density, which were urban characteristics. These findings were consistence with those of a previous study indicating a positive relationship between mortality from these cancers and socioeconomic index of urbanisation [14]. The relationship between mortality from colon and breast cancer and urban residence is plausible considering risk factors of these cancers, such as westernised dietary habits and low birth rate [16].

One mortality cluster for each of colon and breast cancer (MC2 and MC 3, respectively) showed different characteristics from other mortality clusters. It is possible that factors other than those related to urbanisation contributed to the increased mortality in these areas, and further studies are required to elucidate these unique factors. This observation suggests that the factors contributing to the increased mortality in these exceptional areas may be overlooked in conventional ecological studies.

Unlike colon and breast cancer, no dominant societal cluster was observed for lung cancer. The prevalence of smokers was not included in the set of indicators in the present study because municipal data concerning smoking were not available. It is possible that socioeconomic factors used in this study are surrogates of factors related to colon and breast cancers (e.g., dietary habits), while they might not be surrogates of smoking. The higher mortality in Hokkaido prefecture, as identified by MC2, could be explained by the slightly higher smoking rate reported in this area [16]. However, the prefectural data of smoking did not found that other clusters were not related to areas with higher smoking rate [16]. Previous studies showed small variation in male smoking rate and little relationship between smoking rate and regional socioeconomic conditions, and there was no correlation of male smoking rate with lung cancer mortality [17,18]. Thus, it seems that the difference of smoking rate does not thoroughly explain the regional variation in lung cancer mortality rate, although there is no doubt in contribution of smoking to lung cancer, which is the leading cause of cancer deaths in Japanese men [19].

A number of possible contributors to increased mortality from lung cancer in addition to smoking have been reported [20]. Air pollution is an important factor among these possible contributors, and the observation that several clusters of lung cancer were located in metropolitan areas may be explained by the increase in lung cancer due to air pollution. In Okinawa prefecture, local brand cigarettes with a higher tar yield and the prevalence of human papilloma virus infection were suspected to contribute to the increased mortality from lung cancer in this area [21,22]. If multiple factors: *i.e*., smoking, air pollution and other specific local factors, contribute to the regional variation in lung cancer mortality, it is reasonable that no uniform characteristics of mortality clusters were detected in the present study.

We found a similarity of mortality clusters among three types of cancers. Three metropolitan areas (Tokyo, Osaka, and Nagoya) were detected as mortality clusters for all cancers. Urban areas recently show a decrease of the relative health level in Japan, and cancer mortality largely attributes to the decreased health level among urban populations [13-15]. Mortality from several types of cancers seems to be concurrently increased by factors related to urban areas such as health risk behaviour and fewer attendances in cancer screening [23,24]. On the other hand, our findings suggested that the northern part including lots of rural-poor municipalities (SC3) appeared to be another area with higher mortality from some types of cancer. The possible causes of higher mortality in this area should be carefully investigated focusing on differences from those in urban areas.

Several methodological issues about mortality and societal clusters and their relationship should be mentioned. There are several alternative methods for mortality clustering such as Openshaw's and Begas and Newell's methods [25,26]. Although the spatial scan statistic has been widely applied, some possible limitations remain, especially about setting of maximum spatial cluster size and detecting and meaning of the secondary clusters [25,26]. The comparison of SMR mappings and mortality clusters might suggest that municipalities with higher mortality were not necessarily accumulated with circular shape, and thus the non-circular shaped spatial scan statistic [27] could detect more accurate mortality clusters. Due to the use of mortality, instead of incidence, the result in the present study could be influenced by not only cancer incidence but also regional differences in health care qualities and others. The incidence data from such as cancer registration could detect more accurate disease clusters in restricted local areas [28-30], but the incidence data of cancer across the country was not available in Japan.

The societal indicators used in this study were restricted. We used indicators that were demonstrated previously to be critically associated with health level [13,15,31], although some indicators of potential cancer risks may not have been included, especially with regard to health-related behaviour. Second, the societal clustering of municipalities was an important issue in the present study. In contrast to other countries [32-34], as there are no established area classifications or societal indices representing regional characteristics in Japan, we formulated societal indices and classified municipalities by the principle component analysis and the cluster analysis. Different combinations of indicators may result in different figures of societal clusters. Especially, the principle component analysis has been the subject of a variety of criticisms including sensitivity of indicator selection and meaning of the components extracted [35,36], although it has been used to reduce socioeconomic indicators and to obtain one or a few composite index [29,32,37]. In addition, unlike mortality data, societal data were not treated by spatial statistics. Spatial methods such as using population potential [38] instead population density and data smoothing for unstability in the municipalities with small population could contribute to more accurate societal classifications of municipality.

The relation between societal characteristics and mortality was mainly examined using societal clusters and mortality clusters. Societal indices and population density showed the significant relation to mortality according to types of cancer, and they might be more sensitive than societal clusters. The statistical comparisons of societal indices and population density among mortality clusters showed significant differences for most pairs of mortality clusters (data not shown). Thus, the analyses with these variables appeared to be too sensitive to examine homogeneity and heterogeneity among mortality clusters. Since the number of societal clusters was arbitrary in the cluster analysis, an increase of the number of societal clusters would show more complicated variations in the societal characteristics among mortality clusters. Significantly, in the present study even when simple societal clustering was applied, both heterogeneity and homogeneity in societal characteristics among mortality clusters were observed. In addition, the comparison of mortality clusters before and after adjustment for societal characteristics quantitatively supported these heterogeneity and homogeneity.

## Conclusion

The combination of spatial analysis and investigation of the relationships between mortality and societal factors revealed areas in Japan with higher mortality rates and their societal characteristics. The spatial clusters of colon and breast cancer showed homogeneous societal characteristics, with the exception of one cluster. However, the societal characteristics of clusters of lung cancer varied. The homogeneous characteristics of areas with higher mortality rates require strategies across the country or common between higher mortality areas, while exclusive clusters, such as those seen for colon and breast cancer, and variations in societal characteristics for lung cancer imply the need of strategies specific for selected areas with higher mortality.

## Methods

### Study unit and period

Local public entities in Japan are divided into two categories: the first consists of municipalities (*i.e*., cities, towns and villages), while the second consists of prefectures. All districts in the country belong to one of the municipalities and fall within the boundaries of one of the prefectures. Tokyo prefecture (Tokyo Metropolis) includes 23 special wards ("*ku*") in addition to cities, towns and villages. Twelve large cities (cities designated by ordinance), such as Osaka and Nagoya, consist of wards ("*ku*"). In 1995, there were a total of 3372 municipalities (23 Tokyo special ward cities, 127 wards of 12 cities designated by ordinance, 651 cities, 1994 towns and 577 villages) nested within 47 prefectures [39].

The study was performed from 1993 to 1998 during which time several municipalities were annexed or divided, and therefore the aggregated data from these municipalities could not be used. Thus, the final number of municipalities analyzed in the present study was 3360.

### Mortality calculation

In this study, we examined the mortality rates of three high priority cancers: male lung and colon cancer and female breast cancer. Lung and colon cancer were the first and fourth leading causes of cancer death, respectively, in men, and breast cancer was the fourth one in women in the Japanese population in 1995 [20]. The rates of colon and breast cancer have both increased steadily over the last several decades in Japan. Classification was based on the 9th and 10th versions of the International Classification of Diseases (ICD-9 in 1993–94 and ICD-10 in 1995 to 1998): colon cancer, ICD-9 153–154 and ICD-10 C18-C21; lung cancer, ICD-9 162 and ICD-10 C33-C34; and breast cancer, ICD-9 174 and ICD-10 C50 [40,41].

As our focus was on premature mortality, which is more closely related to regional societal characteristics, we examined deaths in the population aged under 75 years old [13]. The numbers of cause-specific deaths by municipality from 1993 to 1998 were compiled. The data regarding deaths in 1995 were excluded to avoid the influence of the Hanshin-Awaji earthquake [14]. Total number of deaths during 5 years was 57,109 for colon cancer, 101,515 for lung cancer, and 32,290 breast cancer. The nationwide age-and cause-specific mortality rates and census municipal age-specific population in 1995 were used as data sources [42]. The aggregated data using macrofiles of the vital statistics were drawn from a database of previous studies [14].

Municipal SMR was calculated and disease mapping was drawn. For calculation of SMR, the hierarchical Poisson regression analysis [13,14,43] was applied since this analysis could correct the unstability in mortality due to heterogeneity of population size: there was marked variation in the population size among municipalities, ranging from a few hundred to a few hundred thousand, and municipalities with a small population showed statistical fluctuation in mortality. The secondary medical care zone (SMCZ), which is defined by prefectural governments for medical care planning according to the Medical Service Law, was used as a higher level. There were 344 SMCZs across Japan in 1995, each of which consisted of neighbouring municipalities and covered a population of 300,000 on average. Bayesian standardized mortality ratio of municipalities was estimated using the iterative generalized least squares (IGLS) and the Markov chain Monte Carlo method [44]. Relative risks (RRs) of societal indices, population density, and societal clusters for cancer morality were estimated using the hierarchical Poisson regression with IGLS. In addition to crude RRs, societal and population density were included in the model to estimate adjusted RRs. For societal clusters, SC1 was used as the reference category. The details of hierarchical Poisson regression are described in previous studies [13,14,44]

### Mortality clusters: spatial scan statistic

The spatial scan statistic was used to detect and evaluate the statistical significance of spatial clusters. The details of the spatial scan statistic were reported previously [4-6,36] and SaTScan ver. 4.0.3  was used for the analysis. The numbers of deaths in each municipality were modelled as Poisson distributions. Under the null hypothesis, the expected number of deaths calculated using age-specific national mortality rates and the age-specific municipal population from the 1995 census [42] was proportional to the indirectly age-adjusted population at risk. An infinite number of circles were superimposed on the map, using the municipal centroid as the centre. The municipal centroid (latitude and longitude) was computed with the map of Japan (geographic coordinate system, GRS 1980; ) using ArcGIS 8.3 (ESRI Japan, Tokyo). The radii of the circles were set to vary continuously from zero to a maximum including at most 10% of the total population at risk, to obtain a certain number of potential clusters. The data for an entire circle contained different sets of neighbouring municipalities, and each circle represented a potential mortality cluster. For each circle, the likelihood was calculated for observing the number of deaths occurring within that circle, and the circle with the maximum likelihood was taken as the primary cluster. The distribution of maximum likelihood under the null hypothesis was evaluated using the Monte Carlo hypothesis testing set with 999 simulations. In addition to the primary cluster, the spatial scan statistic identified the secondary clusters, and ordered them according to the likelihood ratio test statistics. In the present study, secondary clusters were identified using no geographical overlap procedure and those with p-values of less than 0.05 were significant. Mortality clusters were mapped using ArcGIS 8.3 (ESRI, Japan).

### Societal clusters

Based on the findings of previous studies [13,15,31], seven socioeconomic indicators were chosen as potential factors related to mortality (Table 1). These indicators were obtained and calculated using the System of Social and Demographic Statistics consisting of governmental statistics including mainly census data [42]. Unemployment rate reflected the percentage of unemployed persons aged 15–65 years in the total workforce. Educational level reflected the age-adjusted educational level, using the percentage of those who had graduated from college or a higher level among the population aged 20 and over, and was standardised by nationwide sex-and age-specific populations as for standardisation of age-adjusted mortality rate. Income per capita was calculated by aggregating the annual taxable income per household by municipality, and dividing it by the total municipal population.

To reduce the number of variables and identify dimensional societal factors, the principal component analysis with correlation matrix analysis and varimax rotation was performed. The principle components for which the correlation matrix eigenvalues were more than 1.0 were selected as significant dimensions. The component score for the extracted component was assigned to municipalities as a composite societal index: consequently two indices were obtained as shown in Table 1. Then, municipalities were classified into four societal clusters using the K-means cluster analysis with two societal indices and population density (log-transformed). The principle component analysis and the cluster analysis were performed using SPSS 11.0 (SPSS Inc., Chicago, IL, USA).

### Relationships between mortality clusters and societal clusters

The relationships between societal characteristics and mortality clusters identified by the spatial scan statistic were examined by the cross-tabular analysis of mortality clusters and societal clusters.

Furthermore, cluster detections with the spatial scan statistic were performed adjusting for societal indices and population density or societal clusters to determine whether mortality clusters would change before and after adjustment for these variables. The risks of municipalities were calculated using RRs from the hierarchical Poisson regression and used as the adjustment file in the spatial scan statistic [29,44].

## Authors' contributions

YF designed the study, analyzed the data, and drafted the article. MU designed the study and interpreted the results. KN helped to interpret the results and edited the draft. TT supervised the data analysis and writing article.

## References

[B1] Cromley EK, McLafferty SL (2002). GIS and public health.

[B2] Ellioitt P, Wakefield JC, Best NG, Briggs DJ (2000). Spatial epidemiology.

[B3] Rosenberg MS, Sokal RR, Oden NL, DiGiovann D (1999). Spatial autocorrelation of cancer in Western Europe. Eur J Epidemiol.

[B4] Kulldorff M, Feuer EJ, Miller BA, Freedman LS (1997). Breast cancer clusters in the northeast United States: a geographic analysis. Am J Epidemiol.

[B5] Green C, Hoppa RD, Young TK, Blanchard JF (2003). Geographic analysis of diabetes prevalence in an urban area. Soc Sci Med.

[B6] Jemal A, Kulldorff M, Devesa SS, Hayes RB, Fraumeni F (2002). A geographic analysis of prostate cancer mortality in the United States, 1970–89. Int J Cancer.

[B7] Diez Roux AV, Schwartz S, Susser E, Detels R, McEwen J, Beaglehole R, Tanaka H (2002). Ecological variables, ecological studies, and multilevel studies in public health research. Oxford textbook of public health.

[B8] Morgentern H, Rothman KJ, Greenland S (1998). Ecological study. Modern epidemiology.

[B9] Uehara M, Takahashi M, Hoshuyama T, Pan G, Feng Y (2003). Geographical correlation between ambient UVB level and mortality risk of leukemia in Japan. Environ Res.

[B10] Tominaga S, Kuroishi T (1997). An ecological study on diet/nutrition and cancer in Japan. Int J Cancer.

[B11] Aihara H, Iki M (2003). An ecological study of the relations between the recent high suicide rates and economic and demographic factors in Japan. J Epidemiol.

[B12] Nagata C (2000). Ecological study of the association between soy product intake and mortality from cancer and heart disease in Japan. Int J Epidemiol.

[B13] Fukuda Y, Nakamura K, Takano T (2004). Municipal socioeconomic status and mortality in Japan: sex and age difference, and trends of 1973–1998. Soc Sci Med.

[B14] Fukuda Y, Nakamura K, Takano T (2005). Cause-specific mortality differences across socioeconomic position of municipalities in Japan, 1973 to 1998: increased importance of injury and suicide to inequality for ages under 75. Int J Epidemiol.

[B15] Fukuda Y, Nakamura K, Takano T (2004). Wide range of socioeconomic factors associated with mortality among cities in Japan. Health Promot Int.

[B16] Kanda A, Ojima T, Miura Y (2002). Prefectural variation in alcohol consumption, smoking, physical exercise, and obesity and the trend. Koseino Shihyo.

[B17] Fukuda Y, Nakamura K, Takano T (2005). Socioeconomic pattern of smoking in Japan: income inequality and gender and age differences. Ann Epidemiol.

[B18] Asahi S, Watanabe M, Tajimi M (2003). Relationships between prefectural smoking and alcohol consumption rates and cause-specific mortality. Koseino Shihyo.

[B19] Health and Welfare Statitics Association (2002). Kokumin ensei no doko.

[B20] Colditz GA, Atwood KA, Emmons K (2000). Harvard report on cancer prevention volume 4: Harvard Cancer Risk Index. Cancer Causes Control.

[B21] Wakai K, Ohno Y, Genka K (1999). Smoking habits, local brand cigarettes and lung cancer risk in Okinawa, Japan. J Epidemiol.

[B22] Nakazato I, Hirayasu T, Kamada Y, Tsuhako K, Iwamasa T (1997). Carcinoma of the lung in Okinawa, Japan: with special reference to squamous cell carcinoma and squamous metaplasia. Pathology International.

[B23] Fukuda Y, Nakamura K, Takano T (2005). Accumulation of health risk behaviors is associated with lower socioeconomic status and women's urban residence: a multilevel analysis in Japan. BMC Public Health.

[B24] Fukuda Y, Nakamura K, Takano T Reduced likelihood of cancer screening among women in urban areas and with low socioeconomic status: a multilevel analysis in Japan. Public Health.

[B25] Wakefield JC, Kelsall JE, Morris SE, Elliot P, Wakefield JC, Best NG, Briggs DJ (2000). Clustering, cluster detection, and spatial variation in risk. Spatial Epidemiology.

[B26] Waller LA, Gotway CA (2004). Spatial clustering of health events: regional count data. Applied Spatial Statistics for Public Health Data.

[B27] Tango T, Takahashi K (2005). A flexibly shaped spatial scan statistic for detecting clusters. Int J Health Geogr.

[B28] Buntinx F, Gey H, Lousbergh D (2003). Geographical differences in cancer incidence in the Belgian province of Limburg. Eur J Cancer.

[B29] Sheehan TJ, DeChelo LM, Kulldorff M, Gregorio DI, Gershman S, Mroszczyk M (2004). The geographic distribution of breast cancer incidence in Massachusetts 1988 to adjusted for covariates. Int J Health Geogr.

[B30] Kulldorff M, Nagarwalla N (1995). Spatial disease clusters: detection and inference. Stat Med.

[B31] Takano T, Nakamura K (2001). An analysis of health levels and various indicators of urban environments for Healthy Cities projects. J Epidemiol Community Health.

[B32] Krieger N, Chen JT, Waterman PD, Soobader MJ, Subramanian SV, Carson R (2002). Geocoding and monitoring of US socioeconomic inequalities in mortality and cancer incidence: does the choice of area-based measure and geographic level matter?; The Public Health Disparities Geocoding Project. Am J Epidemiol.

[B33] House JM, Lepkowski JM, Williams DR (2000). Excess mortality among urban residents: how much, for whom, and why?. Am J Public Health.

[B34] Morris R, Carstairs V (1991). Which deprivation? A comparison of selected deprivation indexes. J Public Health Med.

[B35] Armitage P, Berrry G, Matthews JNS (2002). Mutivariate methods. Statitical Methods in Medical Research.

[B36] Kachigan SK (1991). Factor analysis. Multvariate Statitical Analysis.

[B37] Folwell K (1995). Single measures of deprivation. J Epidemiol Community Health.

[B38] Middleton N, Gunnell D, Frankel S, Whitley E, Dorling D (2003). Urban-rural differences in suicide trends in young adults: England and Wales, 1981–1998. Soc Sci Med.

[B39] Society for self-government of municipalities (2000). Municipal handbook.

[B40] Ministry of Health and Welfare (1997). Vital statistics 1994.

[B41] Ministry of Health, Labour, and Welfare (1997). Vital statistics 1995.

[B42] Statistics Bureau, Management and Coordination Agency (2000). System of socioeconomic and demographic statistics.

[B43] Leyland AH, McLeod A (2000). Mortality in England and Wales, 1979–1992.

[B44] Rasbash J, Browne W, Goldstein H (2002). A user's guide to MLwiN.

